# Netherton Syndrome: A Case Report and Review of Literature

**DOI:** 10.7759/cureus.3070

**Published:** 2018-07-30

**Authors:** Hafiz M. Kashif Saleem, Muhammad Faizan Shahid, Amir Shahbaz, Atif Sohail, Muhammad Arslan Shahid, Issac Sachmechi

**Affiliations:** 1 Internal Medicine, Allama Iqbal Medical College, Lahore, PAK; 2 Internal Medicine, Jinnah Hospital/Allama Iqbal Medical College, Lahore, PAK; 3 Internal Medicine, Icahn School of Medicine at Mount Sinai Queens Hospital Center, New York, USA; 4 Internal Medicine, Rochester General Hospital, Rochester, USA; 5 Medicine, Nishtar Medical College & Hospital, Multan, PAK

**Keywords:** trichorrhexis invaginata, ichthyosiform erythroderma, atopic dermatitis, netherton syndrome

## Abstract

Netherton syndrome (NS) is a rare autosomal recessive disorder characterized by a triad of congenital ichthyosiform erythroderma (CIE) or ichthyosis linearis circumflexa (ILC), hair shaft abnormalities, and atopic diathesis (elevated serum IgE ). We report a case of a two-year-old boy presented with intractable pruritus, scaling, dry skin and generalized eczematous lesions resistant to atopic dermatitis therapy. Netherton syndrome misdiagnosed as atopic dermatitis due to the presence of eczematous skin lesions and allergic problems. The family counseled about the diagnosis and need of genetic testing for confirmation, but they refused for genetic testing. The patient got treatment with topical corticosteroids and skin moisturizers. There is no cure or satisfactory treatment currently available for NS. Further understanding of the underlying pathophysiology of integumentary changes will lead to more effective treatment. Netherton syndrome should be in the differential diagnosis when characteristic skin manifestation of CIE or ILC, and elevated serum IgE present.

## Introduction

Netherton syndrome (NS, MIM 256500) is a rare autosomal recessive disorder described by Comel (1949) and Netherton (1958). Congenital ichthyosiform erythroderma (CIE) or ichthyosis linearis circumflexa (ILC), hair shaft abnormalities, and atopic diathesis (elevated serum IgE) characterize it [1,2]. Netherton syndrome is usually misdiagnosed as atopic dermatitis due to the presence of eczematous skin lesions and allergic problems. We present a case of NS with intractable skin manifestations, multiple food allergies, initially treated as atopic dermatitis.

## Case presentation

**Figure 1:**
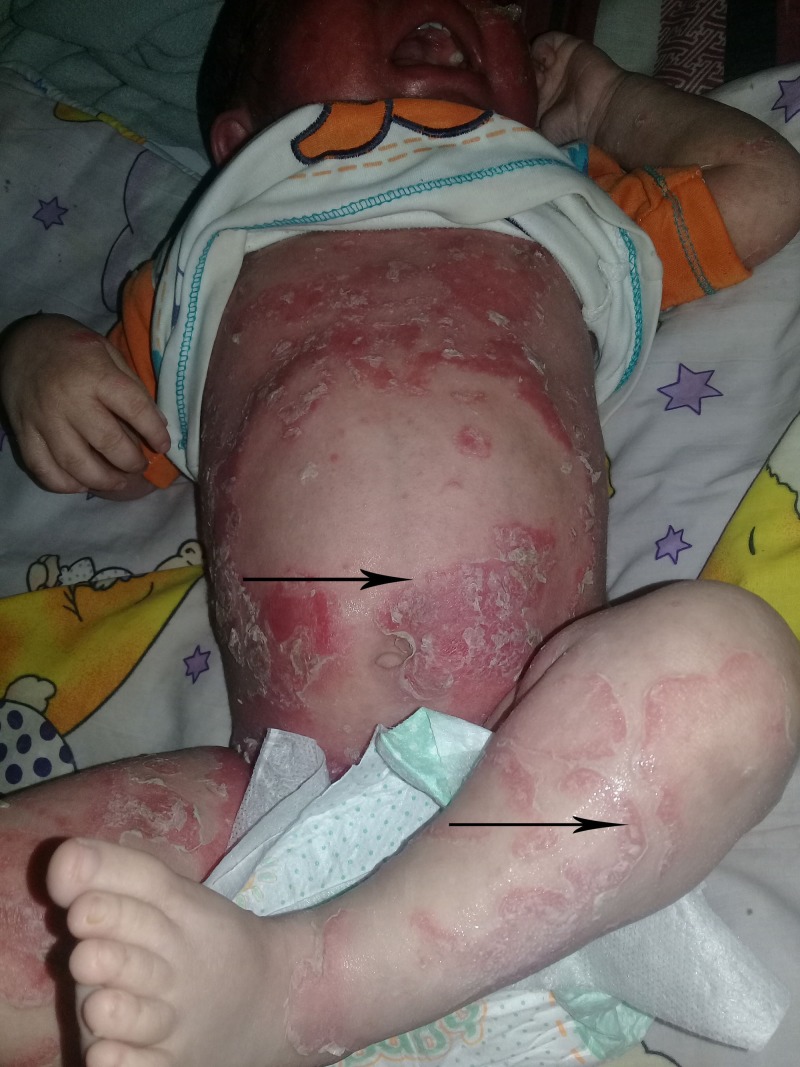
Ichthyosis linearis circumflexia. Erythematous, serpiginous and migratory plaques that have a characteristic of double-edged scale at the margin of the erythema.

**Figure 2:**
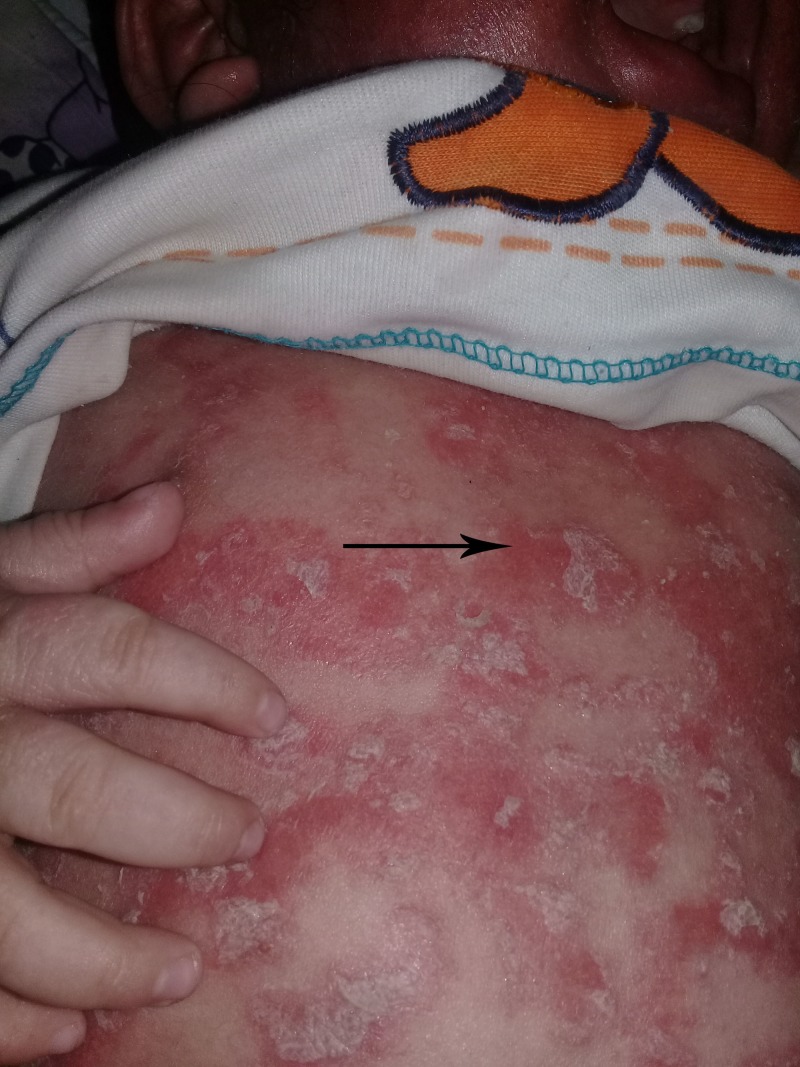
Erythroderma.

**Figure 3:**
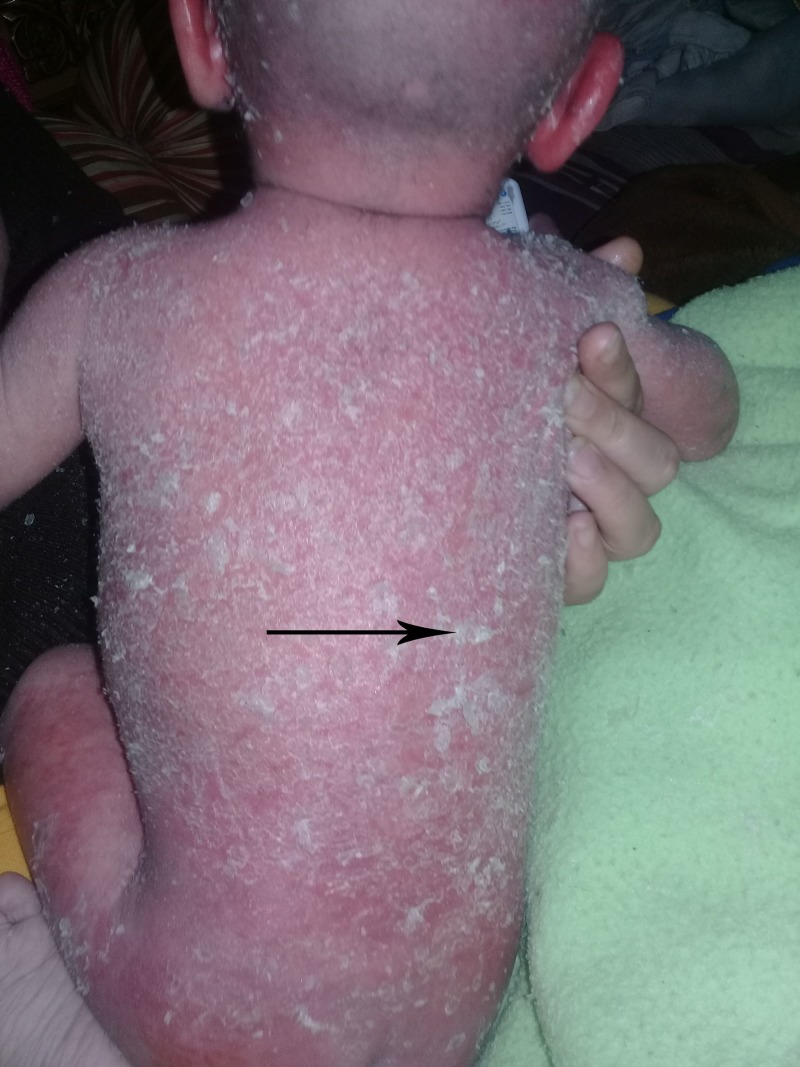
Ichthyosiform erythroderma. Generalized erythroderma and greasy, yellow-to-white scale on back and extremities.

A two-year-old boy referred to our clinic with intractable pruritus, scaling, dry skin and generalized eczematous lesions resistant to atopic dermatitis therapy. Review of his medical record showed he was born at the 37th week of gestation after an uneventful pregnancy to healthy unrelated parents. Shortly after birth, he got treatment for desquamative skin lesions. During the following seven months the desquamation resolved, but ultimately the patient developed generalized, pruritic, erythematous lesions. He got treatment with emollients, topical steroids and tacrolimus creams for severe atopic dermatitis during the next one year. At one year of age, serum IgE levels were 486 IU/ml and 530 IU/ml, respectively. He had no family history of skin disorders. He was allergic to eggs and cow's milk. On physical examination, his skin was dry, and there were erythematous scaly patches on the abdomen, face, and extremities (Figures 1-3).

The eczematous lesions were not typical of atopic dermatitis. The height and weight were normal. Biochemical tests and serum folate, iron, vitamin B12 and zinc levels were normal. He had dry and short scalp hair. The eyebrows and eyelashes were sparse, nails, palms and mucosal surfaces were intact.

Urinary amino acid analysis, immunoglobulins (IgA, IgG, IgM, and IgG), complements (C3, C4) and lymphocyte subset counts (CD3, CD4, CD8, CD14, CD19, CD56) were normal. Serum anti-gliadin IgA and IgG, anti-endomysium IgA, antinuclear antibody (ANA), anti-dsDNA and anti-HIV tests were negative. Thyroid hormone and thyroid autoantibodies were within the normal reference range. The patients had serum eosinophilia and high serum total IgE levels (530 IU/ml). The erythroderma, Ichthyosis linearis circumflexa, elevated IgE along atopic reactions suggest the diagnosis of NS. The patient got treatment with topical corticosteroids and skin moisturizers. The family was counseled about the diagnosis and need of genetic testing for confirmation, but they refused for genetic testing. At six-month follow-up visit, his skin lesion improved but not completely resolved. We keep a close follow-up of the patient.

## Discussion

Netherton syndrome is a rare congenital skin disorder. It is a triad of congenital ichthyosiform erythroderma, trichorrhexis invaginata (TI), and an atopic diathesis [1]. It is characterized as premature desquamation of the stratum corneum and impairment of the skin barrier. Mutations in the serine protease inhibitor (SPINK5) gene located on chromosome 5q31-32 result in increased activity of epidermal proteases which cause desquamation. This condition affects one in 100,000 to 200,000 live births [2]. Congenital ichthyosiform erythroderma is the generalized erythroderma and desquamation present at birth. It evolves into a migratory, serpiginous, erythematous, patches with double-edged scales at the periphery. This Ichthyosis linearis circumflexa waxes and wanes throughout the patient’s life and is accompanied by pruritus [3]. The hair shaft abnormality (Trichorrhexis invaginata) due to invagination of the distal portion of the hair shaft into the proximal portion is pathognomonic. The hairs are typically lusterless, dry, sparse, brittle, and are best observed under trichoscopy or trichogram [4]. In our patient, we did not find TI. Though TI is highly specific, its absence does not exclude the diagnosis of Netherton syndrome [5]. During the neonatal period, hypernatremic dehydration and failure to thrive are common complications. NS is inconsistently associated with delayed growth, mental retardation, aminoaciduria, hypoalbuminemia, immune abnormalities, and enteropathy [6]. Because of the defective skin barrier, recurrent bacterial skin infections are common. Atopic manifestations include atopic dermatitis, urticaria, angioedema and elevated serum IgE [7]. NS is misdiagnosed as atopic dermatitis because of atopic skin involvement and mildly elevated IgE levels [6]. Misdiagnosis happens when specific features, such as the shaft hair abnormality, are not evident. In the presence of positive family history and characteristic cutaneous finding, the diagnosis is straightforward. On the other hand, when the clinical features are atypical, the diagnosis delayed or missed.

The diagnosis is supported by the identification of a germline SPINK5 mutation by DNA sequencing. However, the cost of performing DNA sequencing analysis limits its use in diagnosis [8]. NS is diagnosed upon the presence of allergic manifestations, and one or more of the following: scaling erythroderma, hair shaft abnormalities, history of NS in a sibling, identification of a germline SPINK5 mutation by DNA sequencing. In some patients, the diagnosis of NS relies upon clinical suspicion and findings [9]. Our patient initially got treatment for atopic dermatitis. Eczema resistant to atopic dermatitis therapy, atopic features, and ichthyosiform erythroderma leads us to consider the diagnosis of NS. The elevated IgE and eczema are also present in Wiskott–Aldrich syndrome, but normal platelet count and immune system rule out this diagnosis. The normal level of Zinc, T and B lymphocytes and the absence of opportunistic infections rule out other possible causes of erythroderma including the inborn errors of metabolism and congenital immunodeficiencies.

There is no cure or satisfactory treatment currently available for Netherton syndrome. Topical corticosteroids, topical calcineurin inhibitors, topical retinoids, narrowband ultraviolet B phototherapy, psoralen and ultraviolet irradiation, and oral acitretin are treatment options with varying success. Intravenous immunoglobulin and anti-TNF-α are therapeutic options for severe illness [10-13]. Further understanding of the underlying pathophysiology of skin changes will lead to more effective therapeutic modalities including a possible gene therapy [14].

## Conclusions

Netherton syndrome should be in the differential diagnosis when a child presents with generalized erythema, intractable eczematous lesions, and elevated levels of IgE. Despite the lack of genetic testing, diagnosis can be made when characteristic skin manifestation of CIE or ILC, and elevated serum IgE present.
